# Time-resolved soft X-ray absorption spectroscopy in transmission mode on liquids at MHz repetition rates

**DOI:** 10.1063/1.4993755

**Published:** 2017-08-14

**Authors:** Mattis Fondell, Sebastian Eckert, Raphael M. Jay, Christian Weniger, Wilson Quevedo, Johannes Niskanen, Brian Kennedy, Florian Sorgenfrei, Daniel Schick, Erika Giangrisostomi, Ruslan Ovsyannikov, Katrin Adamczyk, Nils Huse, Philippe Wernet, Rolf Mitzner, Alexander Föhlisch

**Affiliations:** 1 Institute for Methods and Instrumentation for Synchrotron Radiation Research, Helmholtz-Zentrum Berlin für Materialien und Energie, Albert-Einstein-Str. 15, 12489 Berlin, Germany; 2 Institut für Physik und Astronomie, Universität Potsdam, Karl-Liebknecht-Strasse 24-25, 14476 Potsdam, Germany; 3 Department of Physics, University of Hamburg and Center for Free-Electron Laser Science, Luruper Chaussee 149, 22761 Hamburg, Germany

## Abstract

We present a setup combining a liquid flatjet sample delivery and a MHz laser system for time-resolved soft X-ray absorption measurements of liquid samples at the high brilliance undulator beamline UE52-SGM at Bessy II yielding unprecedented statistics in this spectral range. We demonstrate that the efficient detection of transient absorption changes in transmission mode enables the identification of photoexcited species in dilute samples. With iron(II)-trisbipyridine in aqueous solution as a benchmark system, we present absorption measurements at various edges in the soft X-ray regime. In combination with the wavelength tunability of the laser system, the set-up opens up opportunities to study the photochemistry of many systems at low concentrations, relevant to materials sciences, chemistry, and biology.

## I. INTRODUCTION

Near edge X-ray absorption fine structure (NEXAFS) spectroscopy in the soft X-ray regime has for many years been a well-established probe for the local electronic structure in the gas phase, in solution, and in solids.^1^ In recent years, NEXAFS has also been used in pump-probe schemes to detect transient electronic and structural configurations.^2–10^ Direct access to X-ray absorption cross-sections of the investigated sample material is available in a transmission geometry, which is not the case for low yield fluorescence or surface sensitive electron yield detection schemes. While pure thin films of solid samples are regularly used, soft X-ray transmission measurements on solutions have mostly been restricted to cells,^11–20^ where the sample is confined in a sufficiently small volume, separated from the vacuum by membrane windows. Even time-resolved soft X-ray absorption measurements have been performed in this way at repetition rates in the range of 1 kHz.^13,14,19,21^ However, due to slow sample replacement rates within the interaction zone and the risk of membrane damage upon extensive irradiation, cells are disadvantageous in a laser-pump X-ray-probe scheme at MHz repetition rates. Pump-probe transmission measurements at these high repetition rates are performed in tender and hard X-ray regimes using a single free-flowing liquid jet with thicknesses of tens of *μ*m or more.^5,22–31^ Recent advances in liquid jet technology have enabled the possibility of forming liquid sheets with thicknesses small enough to allow for remaining transmission of incident light in the percentage range at soft X-ray photon energies.^32^ It has been shown that the principle of colliding two regular round jets abides the vacuum demands of X-ray spectroscopy methods and provides a complete sample substitution between laser pulses operated at a MHz repetition rate. Therefore, the bunch pattern of modern third generation synchrotrons can be utilized to detect transient changes in the X-ray absorption cross-section of molecules in solution.

In this work, we report on the unique combination of a flatjet sample delivery system with a laser-pump soft X-ray-probe setup yielding high signal-to-noise ratios (S/N) and a temporal resolution on the order of of 50 ps, located at the synchrotron BESSY II (Berlin, Germany). The soft X-ray energy range between 100 and 1000 eV constitutes a very sensitive probe for the local electronic structure of 3*d* transition metal complexes as well as of organic compounds.^17,33–37^ The presented setup is a versatile tool to study systems relevant in different fields of photochemistry like catalysis,^38^ biology^39^ and energy.^40^ As a case study, we present time-resolved NEXAFS data on iron(II)-trisbipyridine $\left[\text{Fe}{\left(\text{bpy}\right)}_{3}^{2+}\right]$ in aqueous solution, where the inter-system-crossing processes following a photo-excitation^41,42^ have been subject to various studies employing a wide range of spectroscopic methods,^43,44^ including X-ray absorption measurements at the ligand^45^ and metal center.^46^ The optical absorption of the system's singlet ground state allows for excitations at various wavelengths. As all excitations induced within this study are at least partly of metal-to-ligand charge-transfer (MLCT) character and therefore result in quintet formation within the 50 ps time resolution, the wavelength tunability of the laser system can be demonstrated. The results of our measurements highlight the excellent S/N achievable with the presented setup, thereby giving leeway to study the photochemistry of dilute systems down to sample concentrations of a few millimoles per liter.

## II. EXPERIMENTAL SETUP

### A. Experimental chamber and detection scheme

**FIG. 1.**
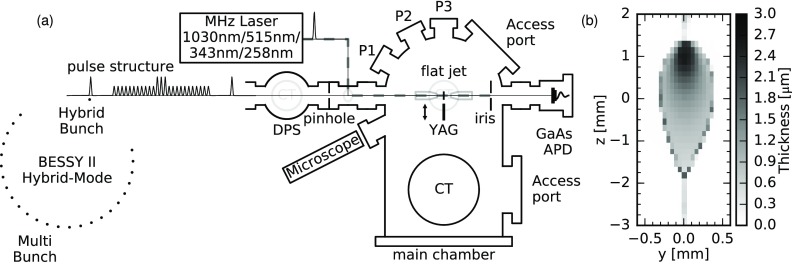
(a) Schematic top view of the experimental layout for static and time resolved NEXAFS measurements of liquid samples. (b) Thickness variation across a water flatjet based on the transmitted intensity at a photon energy of 600 eV and tabulated values for the attenuation length.^51^ Used step sizes were Δy = 50 *μ*m and Δz = 140 *μ*m.

The experimental layout for the static and time-resolved NEXAFS measurements of liquid samples is presented in Fig. 1(a). The liquid sample with the thickness ranging from micrometers to a few hundred nanometers is continuously refreshed in a flatjet system in the main experimental chamber and frozen out in a cooling trap (CT) which is kept at cryogenic temperatures using a liquid nitrogen bath. The CTs together with a 1900 l/s turbo molecular pump (TMP) establish a pressure of 10^−3^ mbar in the main chamber. A pinhole with a diameter of 1.5 mm separates the main chamber from a differential pumping section (DPS) equipped with another CT and TMP (230 l/s), thereby reaching pressures down to mid-10^−8^ mbar in this section. This guarantees acceptable pressure levels in the beamline even during jet instabilities and subsequent pressure jumps. The position of the flat jet is motorized in all three spatial dimensions, and can be controlled with micrometer accuracy.

The transmitted intensity of synchrotron radiation for ground state NEXAFS is measured with a gallium arsenide (GaAs) photodiode. Here, the average diode current is recorded as a measure of transmitted intensity. Scans of photon energy (*E*) dependent intensity with [*I*(*E*)] and without [*I*_0_(*E*)] for the flatjet in the synchrotron beam yield the absorbance of the sample (NEXAFS spectra) $A(E)=-{\text{log}}_{10}[I(E)/I_{0}(E)]$ with the option to extract the absorption coefficients. The measurements presented in this work were performed in the described flexible endstation (not permanently installed at a beamline) at the beamline UE52-SGM located at BESSY II, where the operational energy range of 100–1500 eV enables the probing of soft X-ray absorption edges relevant for organic and inorganic systems. The beamline parameters relevant for our measurements are the small X-ray spot size of (14 × 23) *μ*m^2^ and an energy resolution of E/ΔE > 4000. A more detailed description of the beamline is given in Refs. 47–49. BESSY II provides bunch lengths of currently approximately 50 ps (FWHM) for standard optics, 15 ps (FWHM) for low-*α* optics in the high current mode, and 6 ps (FWHM) for low-*α* optics in the low current mode. Data shown in this manuscript were recorded in BESSY II standard operation. The temporal resolution of 50 ps was estimated from the response function in fits of pump-probe delay scans presented in the latter part of the manuscript.

For time-resolved measurements, we use a commercial fs-laser system from Amplitude Systèmes providing 30 W at its fundamental wavelength of 1030 nm. Harmonics at 515, 343, and 258 nm are generated in a home-built setup to excite the sample. The laser pulse length is on the order of 300 fs FWHM (determined by autocorrelation). Hence, the temporal resolution in the pump-probe experiment is defined by the X-ray pulse length. The transfer of the optical beam to the experiment is actively stabilized to cancel out thermal laser drifts. In this configuration, a stable overlap between the X-rays and the optical spot can be maintained over hours of measurement time if the laser intensity is not altered. At the experiment, the laser beam is focused by a motorized lens pair and consecutively coupled into the vacuum chamber over a piezo-motorized in-vacuum mirror following a path nearly collinear to the X-ray beam. The laser spot size at the sample position is monitored using a spatially resolved detector in an equivalent position outside the vacuum chamber. The spatial overlap of the two beams is established on a fluorescent YAG screen using a far field microscope. Further optimization of the spatial overlap with respect to the transient absorption signal of a sample before each measurement guarantees a comparable overlap of the spots and thus optimized excitation conditions for different measurements. The optical beam is dumped on an iris to reduce stray light or reflections from reaching the avalanche photodiode (APD, active area of 3 × 3 mm^2^, with a min. pulse width of 5 ns FWHM) used for time-resolved measurements. The signal from the optical light is further suppressed below the detection threshold using a 200 nm thick aluminum film in front of the active area of the APD. The temporal overlap between the pulses down to a sub-ns precision is established on an additional APD in the interaction region. The sample position is monitored with a microscope at an angle of 40∘ to be able to precisely place it in the same plane as the YAG screen and thus guarantee the spatial overlap of the beams on the sample.

The signal of the APD is preamplified at the experiment by 20 dB, (1 GHz bandwidth), and recorded with a digital boxcar averager from Zürich Instrument AG allowing to distinguish and record the intensity of X-ray pulses originating from different electron bunches in the fill pattern with high sensitivity. The revolution time of 800 ns for an electron bunch in BESSY II corresponds to the pulse arrival rate of 1.25 MHz. The MHz laser system operated at an integer fraction (*i* ≥ 2) frequency is temporally synchronized with the X-ray pulse pattern to induce excited state dynamics in the investigated system. The synchronization jitter is on the order of a few picoseconds. The temporal delay between the optical and a selected X-ray pulse is set electronically. Usually, the X-ray pulses originating from the hybrid (camshaft) bunch in the gap of the multi-bunch structure in the BESSY II fill pattern are chosen for their higher bunch current. The transmitted X-ray intensity through the excited sample *I_e_*(Δ*t*) at a temporal delay Δ*t* as well as the intensity transmitted (*i* − 1) round trips of the bunch later, i.e., a fresh unexcited sample [*I_g_*, at a temporal delay of Δ*t* + (*i* − 1) × 800 ns], are recorded. This differential NEXAFS measurements of the pumped and unpumped samples cancel out most fluctuations within the experimental conditions, particularly the jet thickness, and thus enables an efficient detection of transient changes. The available pulse energies for different laser harmonics are summarized in Table I. Down to a pulse frequency of 208 kHz, the available laser pulse energy increases inversely to the repetition rate. Below 208 kHz, the pulse energy remains constant and the average output power is reduced. Consequently, down to 208 kHz, a frequency reduction yields significantly higher pulse energies in the fundamental and harmonics, and is beneficial for shorter wavelengths to increase the pumping efficiency with the trade-off of reduced overall statistics. This flexibility in terms of available excitation modes yields a high adaptability of the setup with respect to the attributes of the investigated sample.

**TABLE I.** Typical performance of the laser system used for optical excitation of the samples.

Frequency (kHz)	1250	625	208
Wavelength (nm)	Pulse energy (*μ*J)		
1030 nm	24	48	142
515 nm	8	19	45
343 nm	4	10	20
258 nm	0.2	0.8	6
Spot size (FWHM) (*μ*m^2^)	≥80 × 80		
Pulse length (fs)	300		

### B. The flatjet system

The liquid sample is sprayed into the experimental vacuum chamber via a flatjet system described in detail by Ekimova *et al.*^32^ We will restrict the introduction of the sample injection system to its features relevant for time-resolved NEXAFS measurements.

The flatjet with a thickness from a few hundreds of nanometers to a few micrometers is formed from two round liquid jets colliding in a vacuum [compare Fig. 1(b)]. For the data presented in this work, nozzle diameters of approximately 30 *μ*m were used. The coarse alignment of the jets is achieved by manual adjustments of the micrometer stages on which the nozzles are mounted. The alignment of the nozzles is fine-tuned using in-vacuum piezo-motorized stages. The thickness of the flatjet is dependent on the diameter of the nozzles used to generate the round jets, the flow rate of the liquid sample and the position on the created liquid film. The necessary flow rates to generate a stable flatjet depend on the sample and chosen nozzles, but can be generally assumed to be in the order of a few ml/min. The thickness at the point of interaction with the synchrotron beam can be monitored based on NEXAFS edge-jump measurements.^17,32^ To optimize the X-ray absorption signal with respect to background noise, it is necessary to position the jet with micrometer precision using the motorized manipulator.

For a horizontal jet profile of 500 *μ*m × 1 *μ*m (width × thickness) and a flow rate of 1 ml/min, the sample travels a few tens of *μ*m in the vertical direction within 800 ns. This allows for the investigation of dynamics up to nanosecond timescales, because the sample flow is slow enough for the excited sample to reside in the interaction zone for the inspected time. At the same time, the sample is replenished between two light pulses originating from the hybrid bunch in the BESSY II fill pattern, allowing for the differential detection of the X-ray absorption signal of the sample in its ground and excited states.

## III. RESULTS

By presenting a collection of data acquired with the introduced experimental setup, we highlight its ability to measure static and time-resolved NEXAFS spectra at various absorption edges in the soft X-ray regime for concentrations down to a few millimoles at different excitation wavelengths and fluences. These results represent a benchmark for future experiments on the photochemistry of highly dilute systems.

### A. Transmission NEXAFS of bulk and dilute systems

**FIG. 2.**
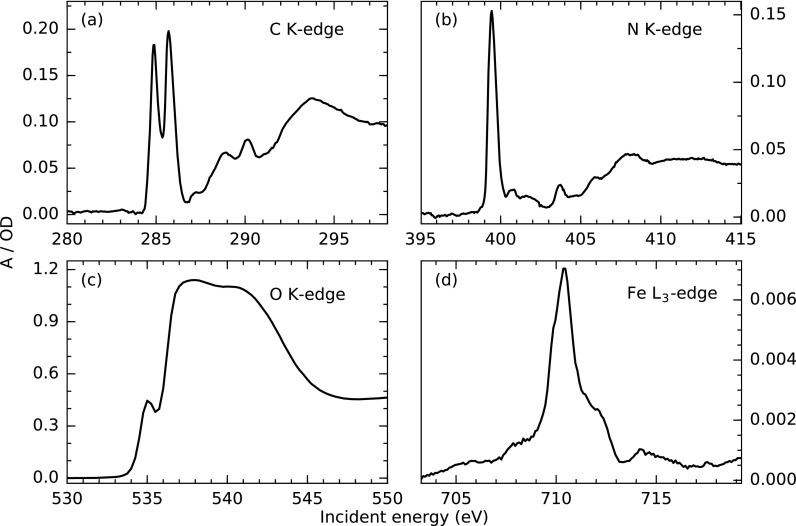
NEXAFS spectra recorded with the flatjet setup at different edges in the soft X-ray energy regime using the GaAs-diode. The average flux from the full BESSY II bunch pattern is used. The (a) C K-edge, (b) N K-edge and (d) Fe L_3_-edge of $\text{Fe}{\left(\text{bpy}\right)}_{3}^{2+}$ (aq, 30 mM; flow rate, 1.8 ml/min) and (c) O K-edge of H_2_O (flow rate, 3.1 ml/min) at jet thicknesses of 2.5, 4.0, 1.3, and 0.8 *μ*m. Incident energy bandwidths were 55 meV (9.8 × 10^10^ photons/s), 95 meV (6.6 × 10^10^ photons/s), 210 meV (3.7 × 10^10^ photons/s) and 45 meV (2.4 × 10^10^ photons/s), respectively. Counting times for the C K-edge and the N K-edge were 4 s per 0.05 eV step, for the Fe L-edge was 30 s per 0.1 eV step and for the O K-edge was 2 s per 0.25 eV step, including measurements of the energy dependent beamline flux *I*_0_(*E*).

Figures 2(a), 2(b), and 2(d) present the transmission NEXAFS spectra of $\text{Fe}{\left(\text{bpy}\right)}_{3}^{2+}$ (aq, 30 mM) at the C K-edge, the N K-edge and the Fe L-edge. The used flow rate for measurements of this complex throughout the manuscript was around 1.8 ml/min. Fine adjustments of the flow rate on the order of ±0.2 ml/min are necessary to achieve an optimized flat jet shape and stability. The spectra qualitatively reproduce previously reported X-ray absorption signatures of the system, although partially measured in non-aqueous solution as well as the solid phase.^45,46,50^ Hence, shifts in energy can be attributed either to the different sample environment or variations between beamline calibrations. We also report, to our knowledge, the first unsaturated spectrum of the water O K-edge from a liquid flatjet displayed in Fig. 2(c). Here, a higher flow rate of 3.1 ml/min was used to achieve a stable sub-*μ*m thin area in the lower part of the flat jet. NEXAFS spectra of highly concentrated samples at tens of molar concentrations ([H_2_O] = 55.5 M) as well as of dilute systems at concentrations down to tens of mM can be acquired in the same experimental configuration. This flexibility originates from the large thickness variation of the flatjet in the vertical direction [depicted in Fig. 1(b)], which allows for optimization of the NEXAFS signal with respect to the used photon energy and sample concentration. Jet thicknesses for the presented measurements are estimated using tabulated X-ray attenuation lengths for a given elemental sample composition.^51^ Since the C K-edge and the N K-edges are energetically below the solvent absorption resonance at the O K-edge, the interaction point was chosen around the top of the flatjet resulting in sample thicknesses of 2.5 *μ*m and 4 *μ*m, respectively, to maximize the absorption of incident light and thus, the contrast of the measurement. As absorption on the O K-edge of the water solvent is significantly higher due to the increase of concentration by three orders of magnitude with respect to the dissolved compound, a much smaller sample thickness of 0.8 *μ*m towards the lower part of the flatjet was chosen in order to avoid saturation effects in the absorption spectrum.

For NEXAFS measurements at edges lying energetically above the absorption edge of the solvent, the sample thickness has to be chosen carefully to optimize the contrast with respect to the absolute signal. A jet thickness of 1.3 *μ*m was chosen for the Fe L-edge NEXAFS measurements of $\text{Fe}{\left(\text{bpy}\right)}_{3}^{2+}$ in Fig. 2(d). Here, approximately 160 ml of sample were used, which was the highest amount of sample for a single edge measurement due to the high impact of solvent absorption. The spectrum [Fig. 2(d)] is generated from averaging 25 individual X-ray absorption measurements involving manipulation of the flatjet position on the order of mm to measure the energy dependent beamline flux in-between the scans. The absorption cross-section of the sample below the Fe L_3_ resonance at 702 eV in these scans is used to determine the thickness of the flatjet for different measurements to quantify the stability of the sample environment throughout the measurements. The individual measurements took 2 min 40 s, and the 25 measurements were recorded within 1 h 30 min. The mean sample thickness extracted for these measurements is 1297 nm with a standard deviation of 3 nm. The maximum and minimum thicknesses are 1293 and 1301 nm, respectively. The highest thickness difference of 5 nm between consecutive scans occurred in correlation with a beamline flux scan requiring the removal of the jet from the beam path and thus a position change by 1 mm. Thickness changes on the few nm scale can also occur randomly during a measurement, without changes of the sample position, due to instabilities of the flow rates in the nozzles. These thickness changes have the largest impact on measurements at photon energies above the main absorption resonances of the used solvent, as the solvent absorption background is strongly altered even upon small thickness changes. Note that this is not the case for the differential measurement of transient absorption changes discussed in the following. Here, changes in the solvent absorption background are reflected in both the excited state and ground state signals making the measurement of transient state spectra and pump-probe delay dependent changes in the X-ray absorption more robust against minor thickness variations of the flatjet.

### B. Time-resolved studies

The system $\text{Fe}{\left(\text{bpy}\right)}_{3}^{2+}$ represents an ideal test case to demonstrate the sensitivity and the tunability of the presented experimental setup as it exhibits a photochemistry on timescales ranging from femtoseconds to a few nanoseconds. We detected signatures of photoexcited states on both the nitrogen K-edge and the iron L_3_-edge.

#### 1. Transient signatures at the N K-edge and the Fe L-edge of iron(II)-trisbipyridine

**FIG. 3.**
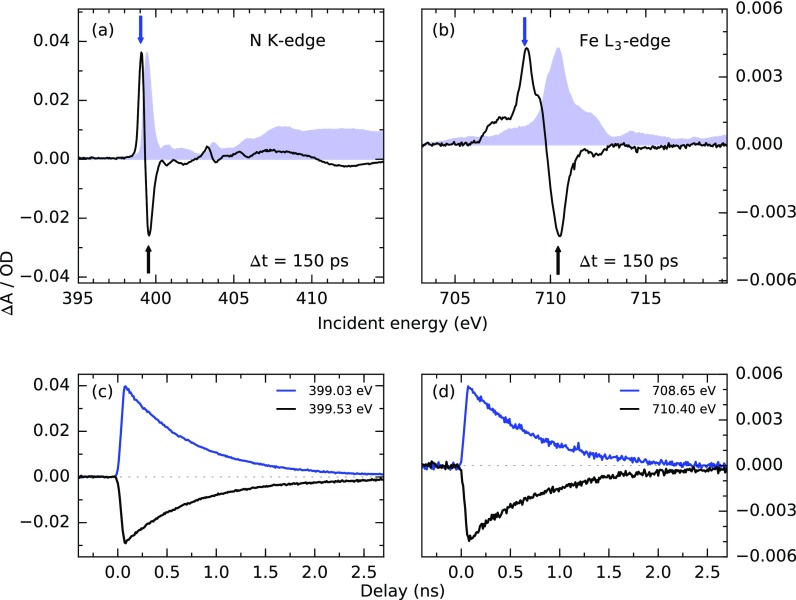
Transient NEXAFS spectra of $\text{Fe}{\left(\text{bpy}\right)}_{3}^{2+}$ (aq, 30 mM) at the N K-edge (a) and the Fe L_3_-edge (b) excited at a repetition rate of 208 kHz using laser pulses at a central wavelength of 343 nm and a power density of about 270 mJ/cm^2^. Static measurements of the N K-edge and the Fe L_3_-edge are shown in blue for comparison. The bandwidth of the incoming X-ray radiation was 130 meV at the N K-edge and 310 meV at the Fe L_3_-edge. At these settings, the flux from the BESSY II hybrid bunch detected with an APD amounts to 2.6 × 10^8^ photons/s (∼1250 photons/pulse) at the N K-edge and 1.5 × 10^8^ photons/s (∼720 photons/pulse) at the Fe L_3_-edge. The noise level of the transient spectra is estimated by the standard deviation of the background level and amounts to 0.1 mOD for a single scan. The counting time for the presented spectra was 1 s per 0.05 eV step at the N K-edge and 4 s per 0.1 eV step at the Fe L_3_-edge. (c) and (d) show the corresponding temporal behavior of the NEXAFS signals at points indicated in the spectra. The counting time for all delay scans was 2 s per 10 ps step.

The electronic and structural responses of dilute $\text{Fe}{\left(\text{bpy}\right)}_{3}^{2+}$ following an optical excitation into the MLCT manifold have been studied by various spectroscopy methods. While the exact excited state pathway in the femtosecond regime is still the subject of an ongoing debate,^44,52^ on a picosecond timescale, the population of the lowest lying quintet state has been unambiguously identified.^41,43^ It exhibits an elongated Fe–N bond compared to the ground state geometry.^53^ At the N K-edge, the dominant spectral signature of this coupled electronic and structural rearrangement in the quintet state has proven to be a shift of the main absorption line towards lower photon energies.^45^ In Fig. 3(a), the transient N K-edge spectrum upon 343 nm excitation is presented, yielding the expected characteristic shift of the absorption maximum. The electrons in the formerly unoccupied e_*g*_ orbitals are reported to donate charge to the ligand *π*-orbitals. Consequently, the shift of the main N 1 s π*-resonance is attributed dominantly to a changed screening of the N 1 *s* core orbital in the quintet state.^45^ The elongation of the Fe-N bond distance in the quintet state coincides with a reduced ligand field splitting of the Fe 3*d* orbitals, as well as an altered coupling between the metal and ligand orbitals.^54^ At the Fe L-edge [compare Fig. 3(b)], this causes the dominant absorption line associated with transitions from the 2*p*_3∕2_ core levels to the unoccupied e_*g*_ states to shift lower in energy and to reduce in intensity. Additionally, an increase in absorption intensity at the low energy side of the main edge corresponding to 2*p*_3∕2_ → t_2__*g*_ transitions is present in the quintet state.

The delay-dependent absorption signals in Figs. 3(c) and 3(d) measured at energies corresponding to the maximal and minimal absorption changes exhibit the same exponential decay constant of (710 ± 20) ps. This value agrees well with the lifetime of the photoinduced quintet state reported by Consani *et al.* and McCusker *et al.* using optical spectroscopy.^42,55^ The transient spectra as well as the delay traces were recorded at a repetition rate of 208 kHz and a laser fluence of 270 mJ/cm^2^ with a background noise level of 0.1 mOD for the individual scans. Under these conditions, a single scan was sufficient to record the spectrum (8 min) as well as the delay-dependent absorption changes (25 min) at the N K-edge. The measurements at the Fe L-edge were performed at a thinner position on the flat jet. In combination with the six-fold lower iron concentration and the lower X-ray absorption cross-section at the Fe L-edge, this yielded an order of magnitude lower X-ray absorption contrast. Therefore, the measurement time was increased to 52 min to achieve a S/N similar to the N-edge spectrum. Measuring the delay-dependent signal with reasonable statistics was still feasible within a single scan of 25 min, however with a reduced S/N.

**FIG. 4.**
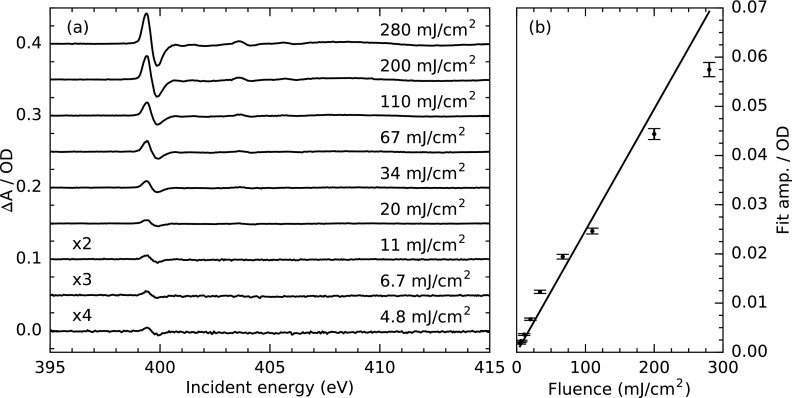
(a) Laser-fluence-dependent transient N K-edge absorption spectra measured at 150 ps delay. The excitation wavelength was 343 nm, and the fluence was varied between 5 and 280 mJ/cm^2^. (b) The first positive amplitude of a two-Gaussian fit for energies below 400.4 eV is plotted against the corresponding excitation fluence. The data were taken under the same measurement conditions as in Fig. 3.

To verify that the transient spectral signatures at high laser fluences are not altered by multi-photon absorption in the sample, Fig. 4 shows the fluence dependence of the transient N K-edge absorption presented in Fig. 3(a). The transient spectra up until 400.4 eV were fitted with the sum of two Gaussians with a positive and a negative amplitude. The amplitude of the lower energy positive Gaussian is plotted in Fig. 4(b) as a function of the used laser fluence. The transient signal strength appears to follow a dominantly linear trend within the accessible fluences. The present deviations from linearity could result from a slight onset of saturation effects as well as from a changed thermal load on the optics upon variation of the laser fluence. Manual adjustments of the spatial overlap between the laser and the X-ray spot were therefore necessary for individual measurements. The latter systematic uncertainty seems to be the most probable reason for the deviations from linearity. This in addition to the fact that the dominant transient signatures are only altered in amplitude upon fluence changes indicates that the recorded transient spectra are not affected by non-linear effects.

Furthermore, the measurements illustrate that transient spectra can still be detected at the lowest measured fluence with a S/N = 10:1 at the peak signal. The high sensitivity for low transient changes is beneficial, especially for excitations using smaller wavelengths with largely reduced available pulse energies.

#### 2. The wavelength tunability of the setup

**FIG. 5.**
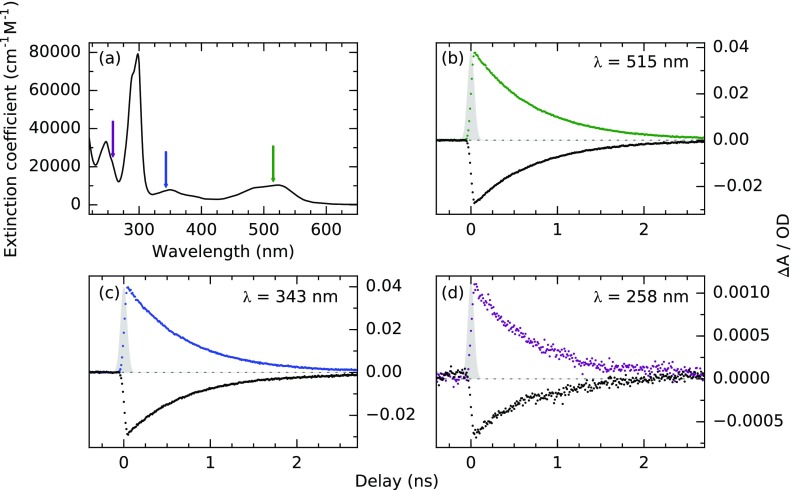
(a) Visible absorption spectrum of $\text{Fe}{\left(\text{bpy}\right)}_{3}^{2+}$ in aqueous solution. (b)–(d) N K-edge picosecond excited state dynamics of $\text{Fe}{\left(\text{bpy}\right)}_{3}^{2+}$ upon excitation into different optical absorption bands. Excitation densities were 210 mJ/cm^2^, 270 mJ/cm^2^, and 50 mJ/cm^2^ for 515, 343, and 258 nm, respectively, at a repetition rate of 208 kHz. The incoming hybrid bunch X-ray flux at energies of 399 eV (colored) and 399.5 eV (black) and at a bandwidth of 130 meV amounts to 2.6 × 10^8^ photons/s (∼1250 photons/pulse). The counting times for delay scans in (b) and (c) were 1 s per 10 ps step and that for the scan in (d) is 6 s per 10 ps.

As indicated in the optical absorption spectrum of aqueous $\text{Fe}{\left(\text{bpy}\right)}_{3}^{2+}$ in Fig. 5(a), the wavelengths of the available laser harmonics correspond to individual optical absorption bands of the system. In Figs. 5(b)–5(d), we present pump-probe delay-dependent N K-edge X-ray absorption changes for the three different excitation wavelengths 515, 343, and 258 nm. We detect qualitatively equivalent transient spectral signatures for all excitation wavelengths at the energies reported in Fig. 3 on the investigated timescale. Therefore, we are able to demonstrate the versatility of the setup in terms of optical excitation wavelengths.

The three measurements exhibit the same single exponential response with a decay time of (710 ± 20) ps. This indicates that the same metastable quintet state is reached by the system on timescales longer than 50 ps as a result of all three optical excitations. The resonances excited with the second and third laser harmonics are known to exhibit MLCT character, while the resonances below 300 nm can be expected to partly correspond to intra-ligand excitations. The independence of the detected dynamics of the exact excitation wavelength indicates that the same spin state is populated by at least a fraction of the excited molecules after 50 ps. For 515 and 343 nm, absorption changes of equal amplitude are induced, potentially due to the similar excitation photon numbers. The detected absorption change for 258 nm is of much lower amplitude, because of the reduced fluence output at the fourth harmonic of the fundamental wavelength and the possibly lower yield of high spin state population.

## IV. CONCLUSION

We presented an experimental setup which enables the acquisition of static and transient soft X-ray transmission NEXAFS measurements of dilute molecular systems in a liquid sample environment. To benchmark the performance of the setup, we demonstrated that the static NEXAFS spectra of both highly concentrated samples, in this case, the O K-edge NEXAFS of liquid water and very dilute systems, like $\text{Fe}{\left(\text{bpy}\right)}_{3}^{2+}$ at a concentration of 30 mM, can be recorded at various X-ray absorption edges. This is achieved by exploiting the high thickness gradient of the liquid flatjet sample delivery system, which allows for precise adjustment of the sample thickness in a wide range. It also provides continuous sample replenishment, which avoids the risk of radiation damage to the sample material and enables in combination with a MHz laser system a differential measurement of optically induced transient X-ray absorption changes. We demonstrated that this quasisimultaneous measurement of excited and ground state absorption, exploiting the BESSY II bunch structure, enables the detection of optically induced absorption changes on the order of 1 mOD. This highly sensitive probe in conjunction with a flexible laser system yields the possibility to investigate a large variety of photoinduced processes in molecular systems with elemental and chemical state selective X-ray absorption spectroscopy in direct transmission.
